# Effect of Alpha-Lipoic Acid in the Treatment of Diabetic Neuropathy: A Systematic Review

**DOI:** 10.7759/cureus.25750

**Published:** 2022-06-08

**Authors:** Saleh A Abubaker, Abdulaziz M Alonazy, Albasseet Abdulrahman

**Affiliations:** 1 Family Medicine, King Faisal Specialist Hospital and Research Centre, Riyadh, SAU; 2 Research, King Faisal Specialist Hospital and Research Centre, Riyadh, SAU

**Keywords:** alpha-lipoic acid, peripheral neuropathy, neuropathy management, diabetic neuropathy, ⍺-lipoic acid

## Abstract

Diabetic neuropathy is a clinical condition that can have a significant impact on quality of life, presenting as numbness, tingling, and burning in the extremities. Current treatment options focus on symptom alleviation and reducing exposure to risk factors as treating the pathophysiological causes of diabetic neuropathy remains a significant challenge. Novel studies have proposed that the use of antioxidants, including alpha-lipoic acid (⍺-lipoic acid), may represent a beneficial intervention for treating neuropathic pain in diabetic patients. This study aims to evaluate the effect of ⍺-lipoic acid in the treatment of diabetic neuropathy and determine its effectiveness in reducing the symptoms of diabetic neuropathy.

To achieve our objective, PubMed, Scopus, and Web of Science databases were screened on March 3, 2022. Randomized controlled trials that investigated ⍺-lipoic acid treatment in diabetes mellitus patients with neuropathic pain and made an appropriate comparison were included. The reduction of neuropathic symptoms was the primary outcome, and the secondary outcome was the incidence of adverse events.

Eight studies comprising 1,500 diabetic patients were evaluated in this systematic review. The findings were inconsistent among the literature concerning the effectiveness of ⍺-lipoic acid in the treatment of diabetic neuropathy, with three trials (37.5%) observing significant improvements in symptoms and five trials (62.5%) not observing any notable results. All studies found ⍺-lipoic acid to be a safe and tolerable intervention, with no reported adverse effects.

The administration of ⍺-lipoic acid may result in symptom reduction and offers a safe and tolerable treatment option. However, there is limited evidence to support the beneficial outcomes of this approach. Further trials are warranted to corroborate or contradict the hypothesis that ⍺-lipoic acid is an effective intervention for the treatment of diabetic neuropathy.

## Introduction and background

Diabetic neuropathy, also referred to as peripheral neuropathy, is a condition that encompasses a wide range of clinical pathologies stemming from peripheral nervous system dysfunction in diabetic patients [1]. The most prevalent presenting symptoms include numbness, tingling, and burning in the extremities, with estimates suggesting a prevalence ranging between 6% and 51% among adult patients with diabetes [2]. Although the exact cause of diabetic peripheral neuropathy is not known, several studies have proposed underlying pathophysiologies, including metabolic, neurovascular, and autoimmune mechanisms. The most widely accepted theory is the induction of oxidative stress in the mitochondria caused by hyperglycemia, which results in hyperglycemic damage. This, in turn, causes damage to the endothelial and neuronal cells, compromising oxygen and nutrient supply to the nerves [3].

Neuropathic pain is a challenge to treat, with most standard analgesics not providing sufficient pain alleviation. The management of diabetic neuropathy, therefore, is divided into four pillars and primarily focuses on addressing underlying risk factors for diabetic neuropathy: (a) pathogenetically orientated therapy, (b) symptomatic therapy, (c) near-normoglycemia, and (d) avoidance of risk factors [4], with near-normoglycemia being the primary aim of treatment. The medication route of treatment mainly incorporates antidepressants, antiepileptics, and opioids, with the first-line therapies being tricyclic antidepressants, serotonin-noradrenaline reuptake inhibitors, and anticonvulsants that target calcium channels. Topical agents, including capsaicin and lidocaine, may also be considered [5]. More recently, antioxidants, including flavonoids and alpha-lipoic acid (⍺-lipoic acid) have been proposed as effective interventions in treating diabetic neuropathy [6].

Given the growing body of evidence concerning the role of ⍺-lipoic acid in the treatment of diabetic neuropathy, this systematic review aims to evaluate the current literature and make recommendations for further research. The focus is on symptom reduction and the incidence of adverse events following administration of ⍺-lipoic acid in this population.

## Review

Methods

Study Design

The primary objective of this systematic review is to assess the effect of ⍺-lipoic acid in the treatment of diabetic neuropathy. Several secondary objectives are also evaluated, including the incidence of adverse effects following ⍺-lipoic acid administration.

Search Strategy

This systematic review was carried out in line with the latest Preferred Reporting Items for Systematic Reviews and Meta-Analyses (PRISMA) guidelines [7], adhering to the 27-point framework of recommended steps [8]. The literature databases PubMed, Scopus, and Web of Science were searched on March 3, 2022, to extract a list of relevant literature for this review. The search terms “(alpha-lipoic acid) AND (diabetic neuropathy) AND (treatment)” were used, alongside their relevant synonyms. Table 1 presents the search terms used in the search strategy, as identified by the Population, Intervention, Comparison, and Outcome (PICO) framework (Table 1).

**Table 1:** Identification of relevant search terms using the Population, Intervention, Comparison, and Outcome (PICO) framework. RCT: randomized controlled trial

	Search terms
Population	“diabetes” OR “diabetic neuropathy” OR “neuropathy” OR “nerve damage” OR “fibromyalgia pain”
Intervention	“alpha-lipoic acid” OR “⍺-lipoic acid” OR “lipoic acid”
Comparison	“standard of care” OR “conventional treatment”
Outcome	“total symptom score” OR “symptoms” OR “pain reduction” OR “pain”
Study design	“randomized controlled trial” OR “RCT”

For study selection, the following inclusion criteria set the parameters for eligibility: (1) a study population composed of diabetes mellitus patients with neuropathic pain, (2) randomized controlled trials (RCTs) investigating ⍺-lipoic acid, (3) and an appropriate comparison was made in the study. Studies were excluded if they were published in a non-English language. Studies were independently identified to be included in the review by a thorough evaluation of the titles and abstracts of the results from the database searches. The final decision to include a study, however, was based on an assessment of the article’s full text. The reference list of the included literature was also reviewed to discover any additional eligible trials for review. Unpublished studies, gray literature, and conference proceedings were excluded (Table 2).

**Table 2:** Inclusion and exclusion criteria.

	Inclusion criteria	Exclusion criteria
Study design	Randomized clinical trials	Observational studies, qualitative studies, books, case reports, gray literature, review articles, conference proceedings
Intervention	Alpha-lipoic acid	Any other antioxidant intervention, other treatment approaches, combination treatment
Population	Type 1 diabetes, type 2 diabetes, diabetes mellitus, adults, peripheral neuropathy	Any non-diabetic population, pediatric populations

Data Extraction

A data extraction form was formulated in line with the PRISMA recommendations [7]. This permitted the extraction of all relevant data from the literature included in this review. This included author(s), year of publication, patient population, intervention, comparison, study period, outcome measures, results, and any conclusions deduced based on the evidence provided regarding the administration of ⍺-lipoic acid among diabetic patients with symptoms of peripheral neuropathy.

Quality Assessment

The authors independently evaluated the quality of each trial using the evaluation form for RCTs developed by the Critical Appraisal Skills Programme (CASP) (http://casp-uk.net). The level of evidence and recommendation for use grades were deduced in line with the Oxford Centre of Evidence-Based Medicine version 2009 (http://cebm.net). The quality of evidence was downgraded if there was a strong risk of bias, imprecision in data collection methods, inconsistency in the findings, indirectness, or publication bias [9].

Results

Identification of the Literature

**Figure 1:**
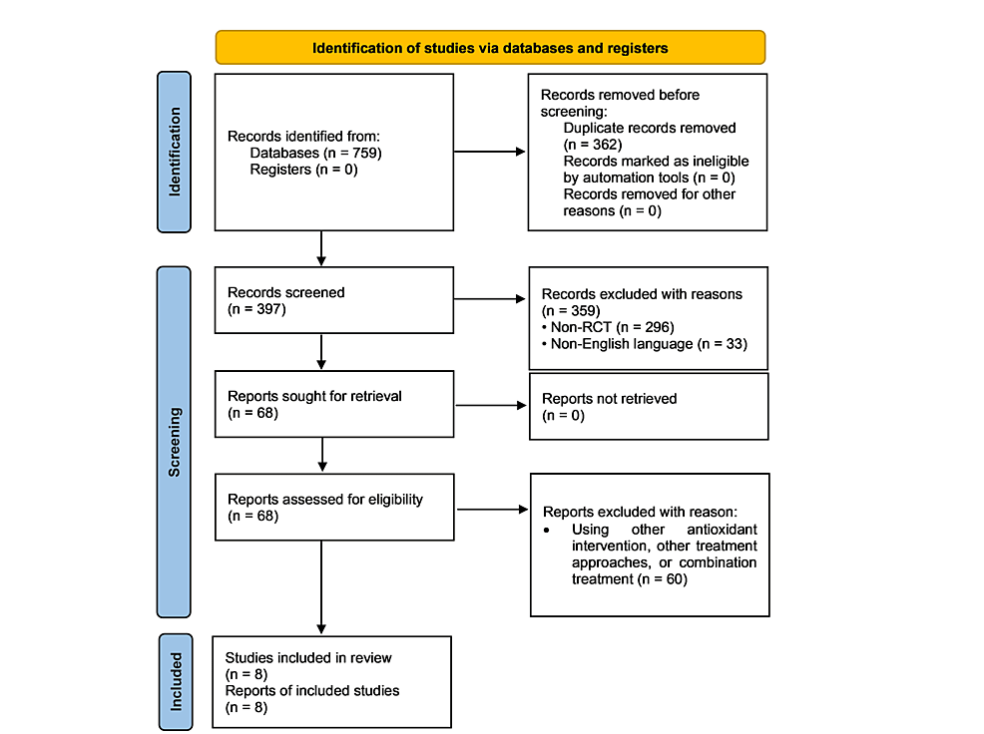
Preferred Reporting Items for Systematic Reviews and Meta-Analyses flow diagram. RCT: randomized controlled trial

Incorporation of the search strategy resulted in 759 studies deemed relevant to the objectives of this systematic review, of which eight articles met the inclusion criteria for eligibility. Figure 1 summarizes the flow of literature at each stage of the screening process, with a total of 68 full-text articles being assessed for eligibility once duplicate studies were removed and the citations were screened against the inclusion and exclusion criteria.

Data Extraction

Eight studies comprising 1,500 diabetic patients were evaluated in this systematic review. All studies followed an RCT design. An overview of the data extraction process is presented in the Appendices.

Population

The populations included in the RCTs had diabetes mellitus. Four studies (50%) exclusively investigated the implications of ⍺-lipoic acid on type 2 diabetic patients with neuropathic pain, one trial (12.5%) solely included type 1 diabetic patients, and three trials (37.5%) included both type 1 and 2 diabetic patients.

Intervention and Comparison

All trials investigated symptom reduction in diabetic patients with neuropathic pain following the prescription of ⍺-lipoic acid; however, the dosage and method of administration varied between studies. Four trials (50%) administered the intervention intravenously (IV), three trials (37.5%) administered the intervention orally, and one trial (12.5%) adopted a combination of both administration methods. The dosages of ⍺-lipoic acid administered ranged from 600 to 1,800 mg/day. The most prevalent dosage administered was 600 mg/day, with this approach being adopted in five trials (62.5%). Two trials (25%) had more than one intervention group, with each division receiving a different dose of ⍺-lipoic acid, varying from 100 to 1,200 mg/day. All trials used an inert placebo as the comparison.

Outcome

The findings observed were inconsistent with each other concerning the effectiveness of ⍺-lipoic acid in the treatment of diabetic neuropathy. Three trials (37.5%) observed a significant improvement in symptoms, including a reduction in the total symptom score (TSS), a reduction in the symptoms of autonomic neuropathy, and improvements in measures of nerve conduction. The remaining five trials (62.5%), in contrast, did not observe any notable results when compared to the baseline or the control group. Moreover, the lack of consistent data collection methods prevented a thorough comparison of the reported outcomes. All studies found ⍺-lipoic acid to be a safe and tolerable intervention, with no reported adverse effects.

Risk of Bias

The Oxford Centre of Evidence-Based Medicine risk of bias assessment is presented in Table 3, with all trials presenting high-quality evidence and moderate recommendations for use given the nature of their design. The CASP tool did not identify any areas for concern, with a low risk of bias being observed across all included studies. This represents the overall strength of this systematic review and its findings.

**Table 3:** Oxford Centre of Evidence-Based Medicine risk of bias.

Author (year)	Quality of evidence	Recommendation for use
Gilron et al. (2021) [10]	High	Moderate
El-Nahas et al. (2020) [11]	High	Moderate
Won et al. (2020) [12]	High	Moderate
Liu et al. (2007) [13]	High	Moderate
Tankova et al. (2004) [14]	High	Moderate
Reljanovic et al. (1999) [15]	High	Moderate
Ziegler et al. (1999) [16]	High	Moderate
Ziegler et al. (1997) [17]	High	Moderate

Discussion

This systematic review aimed to evaluate the effectiveness and safety of ⍺-lipoic acid in the treatment of neuropathic pain in diabetic patients. We observed that the administration of ⍺-lipoic acid offers an alternative intervention for the treatment of diabetic neuropathy and is a safe and tolerable approach. However, significant findings were only observed in three trials included in this review, with the remaining literature failing to present any notable evidence. Hence, further trials are warranted to corroborate or contradict the hypothesis that ⍺-lipoic acid is an effective intervention for the treatment of diabetic neuropathy.

A review by Vallianou et al. proposed that ⍺-lipoic acid achieves its symptom alleviation by delaying or reversing peripheral diabetic neuropathy using its antioxidant properties. In particular, ⍺-lipoic acid increases glutathione, an endogenous antioxidant involved in antioxidant defense, nutrient metabolism, and the regulation of cellular events [18,19]. Moreover, the literature highlights that glutathione deficiency contributes to oxidative stress, which plays a crucial role in the pathogenesis of diabetic neuropathy [20]. However, the literature does suggest that the effect of ⍺-lipoic acid is greater when used with conventional treatment. A trial by Karalis et al. investigating the effect of ⍺-lipoic acid as a combination treatment on diabetic peripheral neuropathy in 148 type 2 diabetics provided evidence in this respect. The peripheral neuropathy development score significantly decreased among all participants following the administration of a combination of treatments, including ⍺-lipoic acid at 600 mg/day, gliclazide, sodium-glucose-linked transporter 2 inhibitors, metformin, and glucagon-like peptide 1 analogs. Treatment was monitored over eight months. Collectively, these findings highlight the beneficial impact of ⍺-lipoic acid when used as part of a combination regime in patients with type 2 diabetes experiencing neuropathic pain [21].

Han et al. conducted a systematic review and meta-analysis of RCTs that investigated nerve conduction in diabetic patients with peripheral neuropathy. The findings of this review supported and corroborated the evidence presented in our review, observing significant improvements in only a handful of the included studies. Moreover, this review also reported on the lack of consistent methodologies and poor methodological quality, which resulted in weak evidence being presented [22].

Limitations

Despite the strengths of this review, such as only including RCTs, there were several limitations that must be considered. First, we exclusively investigated the implications of ⍺-lipoic acid as a standalone treatment for diabetic neuropathy, excluding any trial that incorporated this intervention as a combination treatment. This may provide a rationale for the limited evidence observed concerning the sole beneficial effect of ⍺-lipoic acid on neuropathic pain. Previous trials, such as that of Wang et al., found significant improvements in diabetic peripheral neuropathy symptoms when ⍺-lipoic acid was combined with epalrestat [23]. Future reviews should consider this limitation and compare the patient outcomes of ⍺-lipoic acid alone and in combination with current treatments for diabetic neuropathy. Second, the lack of consistent data collection methods across the included trials negated a meta-analysis from being conducted; hence, we were unable to quantitively evaluate the effect of ⍺-lipoic acid on the occurrence of diabetic neuropathy.

## Conclusions

Based on the majority of evidence from this systematic review, we conclude that the use of ⍺-lipoic acid alone provides no significant improvement in the treatment of neuropathic pain among diabetic patients. However, it is considered a safe and tolerable treatment option that may result in some neuropathic symptomatic reduction. Further future trials could incorporate this intervention as a combination with current treatments for diabetic neuropathy considering its notable efficacy and good safety profile.

## Appendices

Supplementary materials

**Table 4:** Data of studies included in this review.

	Author (year)	Population	Intervention	Comparison	Study period	Outcome measures	Results	Conclusion
[10]	Gilron et al. (2021)	24 patients (mean age: 57 years)	Alpha-lipoic acid (ALA) capsules at a median dose of 1,663 mg/day	Inert placebo capsules	Two treatment periods of five-week duration	Treatment-emergent adverse effects, pain intensity	No significant differences (p = 0.7) in pain intensity or adverse effects were observed	There was no evidence of the beneficial impact of ALA in the treatment of diabetic neuropathy
[11]	El-Nahas et al. (2020)	200 patients	Oral 600-mg ALA capsules	Inert placebo capsules	Six months, outcome measures assessed at months 1, 3, and 6	Vibration perception threshold, neurological symptom score, neurological disability score, pain intensity	ALA-treated patients had significantly improved outcome measures (p < 0.05) in all parameters	Oral 600-mg ALA twice-daily treatment over a 6-month period was effective, safe, and tolerable in the treatment of diabetic neuropathy
[12]	Won et al. (2020)	100 patients with type 2 diabetes	ALA (600 mg/day)	GLA (320 mg/day)	12-week treatment period	Mean change in pain intensity, TSS	No significant differences were observed between groups at the end of the study period (p > 0.05)	ALA and GLA yielded similar results in terms of reducing pain intensity in patients with diabetic neuropathy
[13]	Liu et al. (2007)	95 type 2 diabetic patients	ALA (600 mg/day) in normal saline 250 given by IV drip infusion	Radix salvia 20 mL given by IV drip infusion	14 days	Fasting glucose, fasting insulin, supersensitive C-reactive protein, HbA1c, TSS, Michigan neuropathy screening instrument	TSS of numbness, stinging sensation, and burning sensation were significantly reduced after 2 weeks of ALA (p < 0.01)	ALA effectively improved the sensitivity symptoms of diabetic neuropathy patients
[14]	Tankova et al. (2004)	75 patients with type 1 diabetes and different forms of autonomic neuropathy	ALA IV (600 mg/day)	Inert placebo	60 days	Ewing’s tests, laboratory parameters of oxidative stress	Significant improvements were observed in the symptoms and signs of autonomic neuropathy in the ALA group. Changes were also observed in the laboratory parameters of oxidative stress, although these were not significant	ALA may be an effective drug in the treatment of different forms of diabetic neuropathy
[15]	Reljanovic et al. (1999)	169 patients with type 1 or type 2 diabetes and symptomatic diabetic polyneuropathy	Oral ALA: six tablets/day at 200 mg, three tablets/day at 200 mg, thee tablets of a placebo, and six tablets of placebo	Inert placebo	24 months	Neuropathy disability score	Statistically significant changes were observed at 24 months between ALA and comparative groups concerning measures of nerve conduction (p < 0.05). No significant differences between the groups were observed concerning the neuropathic disability score (p > 0.05)	Oral ALA may have a beneficial effect on several measures of nerve conduction; however, further studies are warranted to determine if this intervention reduces the neuropathy disability score and overall symptoms
[16]	Ziegler et al. (1999)	509 outpatients with type 2 diabetes and symptomatic polyneuropathy	ALA IV (600 mg/day) for three months followed by oral ALA, 600 mg three times daily	Inert placebo	7 months	TSS for neuropathic symptoms, neuropathy impairment score	No significant differences were observed between groups at the end of the study period (p = 0.447). Favorable effects were observed on neuropathic deficits	ALA and the placebo yielded similar results in terms of reducing the pain intensity of patients with symptomatic polyneuropathy. Future long-term trials should be conducted to assess the impact of ALA on neuropathic deficits as opposed to symptom reduction
[17]	Ziegler et al. (1997)	328 patients with non-insulin-dependent diabetes mellitus and symptomatic peripheral neuropathy	ALA IV at either 1,200 mg, 600 mg, or 100 mg	Inert placebo	Three weeks	TSS, Hamburg pain adjective list	A significant decrease in symptoms was observed across all intervention groups. The ALA 600 mg group had significantly lower symptom scores compared to the placebo group (p < 0.05)	ALA IV at 600 mg/day over a three-week period is a safe and effective intervention to reduce symptoms of diabetic peripheral neuropathy
